# Genome-wide association study and transcriptome analysis reveal key genes controlling fruit branch angle in cotton

**DOI:** 10.3389/fpls.2022.988647

**Published:** 2022-09-21

**Authors:** Panxia Shao, Yabin Peng, Yuanlong Wu, Jing Wang, Zhenyuan Pan, Yang Yang, Nurimanguli Aini, Chunping Guo, Guangling Shui, Lei Chao, Xiaomin Tian, Qiushuang An, Qingyong Yang, Chunyuan You, Lu Lu, Xianlong Zhang, Maojun Wang, Xinhui Nie

**Affiliations:** ^1^Key Laboratory of Oasis Ecology Agricultural of Xinjiang Production and Construction Corps, Agricultural College, Shihezi University, Shihezi, Xinjiang, China; ^2^National Key Laboratory of Crop Genetic Improvement, Hubei Hongshan Laboratory, Huazhong Agricultural University, Wuhan, Hubei, China; ^3^College of Informatics, Huazhong Agricultural University, Wuhan, Hubei, China; ^4^Institute of Nuclear Technology and Biotechnology, Xinjiang Academy of Agricultural Sciences, Urumqi, Xinjiang, China; ^5^Cotton Research Institute of the Shihezi Academy of Agriculture Science, Shihezi, Xinjiang, China

**Keywords:** plant architecture, cotton, fruit branch angle, GWAS, transcriptome analysis

## Abstract

Fruit branch angle (FBA), a pivotal component of cotton plant architecture, is vital for field and mechanical harvesting. However, the molecular mechanism of FBA formation is poorly understood in cotton. To uncover the genetic basis for FBA formation in cotton, we performed a genome-wide association study (GWAS) of 163 cotton accessions with re-sequencing data. A total of 55 SNPs and 18 candidate genes were significantly associated with FBA trait. By combining GWAS and transcriptome analysis, four genes underlying FBA were identified. An FBA-associated candidate gene *Ghi_A09G08736*, which is homologous to *SAUR46* in *Arabidopsis thaliana*, was detected in our study. In addition, transcriptomic evidence was provided to show that gravity and light were implicated in the FBA formation. This study provides new insights into the genetic architecture of FBA that informs architecture breeding in cotton.

## Introduction

Cotton (*Gossypium* spp.) is an important economic crop cultivated worldwide and the largest source of textile fiber (Chen et al., 2007). To increase planting density and facilitate mechanical harvesting, compact plant architecture is required for cotton cultivars grown in Xinjiang Uygur Autonomous Region, which is currently the largest cotton growing area with the highest yield in China. Ideal plant architecture can improve the leaf area index, photosynthetic efficiency of population and grain yield in crops, which suggest that plant architecture is closely related to crop production and breeding (Wang and Li, 2008a). Among plant architecture traits in crops, the branch angle is closely related to planting density (Zhao et al., 2014), disease resistance (Wang and Li, 2008b) and photosynthetic efficiency (Pendleton et al., 1968), and ultimately affects plant productivity and grain yield in many crops. Therefore, the branch angle is one of the important factors for plant architecture, which refers to the angle between main stem and branch (Bai et al., 2012).

The regulation mechanism of branch (tiller or leaf) angle is complex, and many environmental signals including light and gravity play an essential role in branch angle formation. To date, many gravity-related genes associated with branch angle have been identified. For instance, shoot gravitropism (SGR) family change the branch angle by attenuating gravity perception in *Arabidopsis* (Fukaki et al., 1996; Yamauchi et al., 1997). *SGR9* attenuates gravitropism and increases branching angle by affecting the interaction between actin filament (AF) and amyloplasts (Nakamura et al., 2011). *SGR5* is thought to sense gravity in endodermal cells (Morita et al., 2006), and *Loose Plant Architecture1* (*LAP1*), which is the functional ortholog of the *SGR5* gene in *Arabidopsis*, negatively regulates leaf angle by controlling the adaxial growth of tiller node and lamina joint in rice (Wu et al., 2013). Many studies have shown that LAZY also play a key role in regulating the plant branch angle *via* the gravitropism. In *Arabidopsis*, *AtLAZY* genes control inflorescence branch angle by coupling gravity sensing (Yoshihara and Spalding, 2017). In rice, *LAZY1* and *LAZY2* controlled tiller angle by regulating shoot gravitropism and starch biosynthesis in gravity-sensing cells, respectively (Li et al., 2007; Huang et al., 2021b). *CsLAZY1* also played an important role in regulating shoot gravitropism in tea plants (Xia et al., 2021). Light is also one of the most important environmental signals regulating plant growth and development. Light can also affect rice tiller angle by regulating the level of the *OsPIL15*, depending on the main photoreceptor phytochrome B (Xie et al., 2019). In the study of light morphogenesis, PIF3 (phytochrome interacting factors, PIF) negatively regulates chlorophyll biosynthesis and acts on the expression of photosynthesis related genes such as phytochrome A (PHYA), resulting in a lower ratio between red light and far-red light of the environment (Shina et al., 2009), and EID1 can shift the response of the PHYA, acting synergistically in this process (Dieterle et al., 2001).

The polar auxin transport plays an important role in branch angle formation, shoot elongation, gravitropism and lateral shoot formation. Several branch angle formation genes related to auxin transport were identified. An auxin signaling gene *BnaA3.IAA7*, which encodes an Aux/IAA protein, reduced branch angle in rapeseed (Li et al., 2019a). Gravity stimulation polarizes auxin efflux carriers PIN-FORMED3 (PIN3) to the bottom sides of endodermal cells, auxin accumulation polarizes in adjacent tissues at the lower side of the stimulated organ, where auxin induces cell elongation and, hence, organ bending (Rakusova et al., 2016). In addition, the auxin response factors (ARFs) can regulate leaf angle by increasing adaxial cell division and regulating secondary cell wall biosynthesis of lamina joints in rice (Zhang et al., 2015; Huang et al., 2021a). *SMALL AUXIN UPREGULATED RNA 10* (*SAUR10*) is specifically expressed at the abaxial side of the branches and this localized activity is influenced by hormones, light conditions and the *Arabidopsis* MADS-domain factor FRUITFULL, which also has an effect on branch angle formation (Bemer et al., 2017).

Recently, with the development of high-density SNP genotyping, genome-wide association study (GWAS) has been gradually applied to the study of complex traits in crops. GWAS has been performed on tiller angle in rice (Dong et al., 2016), leaf angle in sorghum and maize (Tian et al., 2011; Maldonado et al., 2019; Zhi et al., 2022), and branch angle in rapeseed (Sun et al., 2016; Li et al., 2017). *OsbHLH153* and *OsbHLH173* were identified through GWAS in regulating leaf angle and overexpression in plants increased rice leaf angle, providing new insights for ideal rice architecture breeding (Dong et al., 2018). The application of GWAS to the branch, tiller and leaf angle provides a guide for dissecting the genetic basis and studying complex traits.

The proper fruit branch angle (FBA) can be helpful for adaptation to mechanistic harvesting and yield improvement. Although some QTLs or genes regulating FBA in cotton have been previously identified through parental mapping populations and natural populations (Song and Zhang, 2009; Li et al., 2014, 2015; Su et al., 2018), the effective dissection of the genetic basis of FBA are still not comprehensive enough. Therefore, the phenotype data of FBA in 163 *Gossypium hirsutum* cultivars with wide variations were collected in Korla and Shihezi, Xinjiang in this study. Through GWAS, genetic loci and key candidate genes related to FBA were explored. Moreover, we performed transcriptome sequencing of two *G. hirsutum* cultivars with significant FBA difference, and identified key regulatory genes and pathways, which laid a foundation for gene function verification, molecular mechanism study of FBA formation and cotton genetic improvement in the future.

## Materials and methods

### Plant materials and sample processing

The 163 upland cotton germplasm resources, which were composed of 152 white-fiber cultivars (Nie et al., 2020) and 11 brown-fiber cultivars (Supplementary Table S1), were planted in multiple environments including E1 (Korla, Xinjiang Uygur Autonomous Region, China, in 2018; 86.06°E, 35.05°N); E2 (Korla, Xinjiang Uygur Autonomous Region, China, in 2019; 86.06°E, 35.05°N); E3 (Shihezi, Xinjiang Uygur Autonomous Region, China, in 2018; 85.94°E, 44.27°N); and E4 (Shihezi, Xinjiang Uygur Autonomous Region, China, in 2019; 85.94°E, 44.27°N). Then, these germplasm resources were subjected to phenotyping and genotyping analysis for GWAS analysis. Two upland cotton cultivars, Xinluzhong45 (Z45) with large-FBA and Xinluzao2 (Z2) with small-FBA were used for transcriptomic analysis. The axillary buds were collected in the pre-squaring stage and the fruit branches were 1 cm connecting with the main stem from the 3rd to 5th fruit branches, which were divided into upper side and lower side and collected in the squaring stage for three individual plants of each material. For each stage, three samples were pooled together for each of the three biological replicates of RNA extraction. These samples were frozen immediately in liquid nitrogen and stored in a freezer at −80°C.

### Anatomical observation on fruit branch

In squaring stage, we connected the main stem from the 3rd to 5th fruit branches, which were divided into upper side and lower side for anatomical observation. The plant samples were immersed in FAA stationary solution (70% alcohol: formaldehyde: acetic acid, 18:1:1 v/v). Samples were rehydrated in two changes of xylenes for 20 min and 75% alcohol for 5 min, rinse with running water. Subsequently put samples into safranin O staining solution for 15–30 s and dehydrated in a gradient alcohol series (50, 70, and 80% alcohol for 3–8 s), after using plant solid green staining solution staining 6–20 s. Finally, put samples into three cylinders of xylene for 5 min each time, mount the tissue section with neutral balsam. A panoramic MIDI automatic digital slide scanner (3DHISTECH Ltd., Budapest, Hungary) was used for image processing and quantification.

### Phenotypic data collection and statistical analysis

In the field, FBA was measured as the angle between the cotton stem and fruit branch and was recorded for the third to fourth fruit branch from the top to bottom and 10 typical plants were selected in 4 environments. The average value from 10 plants represents the phenotypic data of each accession and FBA across two replicates within 1 year was used for GWAS. A digital protractor was employed to measure the angle between the main stem and fruit branch of cotton.

Spss25.0 was used for the statistical analysis of FBA. Correlation analysis of FBA for the association panel across different environments was performed in R software. QTL IciMapping 4.2 was used for analysis of variance (ANOVA) and broad-sense heritability. The broad-sense heritability was estimated according to the following equation: $H^{2}=V_{G}/\left(V_{G}+\frac{1}{e}V_{GE}+\frac{1}{re}V_{\varepsilon }\right)$where, $V_{G}$ $V_{GE}$, Vε, and r represent the genetic variance, the interaction variance between genotypes and environments, the error variance, the number of replicates within each environment and the number of environments, respectively.

### Genotypic data analysis

For each accession, a young leaf was collected from the plant, and genomic DNA was extracted for construction of a paired-end sequencing library to perform 10× genomic coverage re-sequencing with the Hiseq 2000 platform (Illumina, Inc., San Diego, CA, United States). Clean reads derived from 163 accessions were aligned against the *G. hirsutum* reference genome TM-1 (WHU_updated v1) (Huang et al., 2020) using BWA, version 0.7.10 (Li and Durbin, 2009). After alignment, SNP calling on a population scale was performed with the Unified Genotyper method by using the Genome Analysis Toolkit (GATK, version v3.1) (McKenna et al., 2010). Subsequently, high-quality SNPs with minor allele frequency (MAF) more than 0.05 were retained for further analysis. The population genetic structure was examined *via* an exception maximization algorithm, as implemented in the program Admixture (Pritchard et al., 2000). The number of assumed genetic clusters K ranged from 1 to 10, with 10,000 iterations for each run. We also conducted principal component analysis (PCA) to evaluate genetic structure by using GCTA software (Li and Durbin, 2010). The phylogenetic tree was constructed by using TreeBestv1.9.2 software. The PopLDdecay was used to calculate LD decay.

### Genome-wide association study

The association panel containing 163 samples, has a total of 2,499,987 SNPs (MAF ≥ 0.05). We used the sliding window method with default settings for 50 bp window size and 10 bp step size to view the SNPs across the whole genome. The threshold line of association analysis was set at −lg (1/*N*) (*N* is the valid of SNP markers). The best linear unbiased prediction (BLUP) and individual environment value was used as the phenotype for association analysis. Genome-wide association analysis was performed between SNPs and trait using the genome-wide efficient mixed-model association (GEMMA) (Zhou and Stephens, 2012) with a mixed linear model (MLM), and the optimal structure subgroups (*Q*) and kinship (*K*) were employed to correct stratification.

### RNA isolation and transcriptome sequencing

Total RNA was extracted separately from each sample using an RN38 EASYspin plus Plant RNA kit (Aidlab Biotech, Beijing, China). RNA integrity was assessed using the RNA Nano 6000 Assay Kit of the Bioanalyzer 2100 system (Agilent Technologies, CA, United States). The libraries were sequenced by Novogene (Novogene, Tianjin, China) with an Illumina HiSeq (Illumina, CA, United States) system. To gain more reliable data, reads from each data set with more than 10% N bases and low quality (*Q* ≤ 20) reads with more than 50% bases were removed (Chen et al., 2018). Eventually, the clean reads were mapped to the TM-1 reference genome (*G. hirsutum*, WHU_updated v1) (Huang et al., 2020) by Hisat2 v2.0.5. To calculate gene expression, the number of mapped clean reads for each gene was counted and normalized into Fragments Per Kilobase of transcript sequence per Millions (FPKM).

### Differential gene expression and functional enrichment analysis

DESeq2 R package (1.20.0) (Love et al., 2014) was used to analyze differentially expressed genes (DEGs) to correct for multiple testing, and the false discovery rate (FDR) was calculated to adjust the threshold of the *p*-value by using the Benjamini and Hochberg’s method. Genes with a minimum 2-fold difference in expression, |log2FoldChange| > 1 and FDR < 0.05 were considered as DEGs.

Gene Ontology (GO) enrichment analysis of differentially expressed genes was implemented by the clusterProfiler R package. The *p*-value of the GO enrichment analyses were adjusted using the Benjamini and Hochberg’s method, and FDR < 0.05 was chosen as the threshold value for determining significantly enriched GO terms. Based on all expressed genes, PCA was carried out to explain the relationship among all samples.

### Real-time quantitative PCR validation

In order to validate the RNA-sequencing results, the qRT-PCR were carried on candidate genes. The specific primers were designed by using online tools: Primer-BLAST.^1^ The specific primers for the selected genes and the actin gene (UBQ7) are listed in Supplementary Table S2. The qRT-PCR was performed on C1000 Touch Thermal Cycler using SYBR Green qPCR Super Mix (Transgen Biotech, Beijing, China). The cotton UBQ7 gene was used as an internal standard to calculate relative fold differences based on comparative cycle threshold (2^−ΔΔCt^) values (Livak and Schmittgen, 2001). The reaction mixture of qRT-PCR was as follows: 0.5 μl of each primer was added into 10 μl of SYBR Green qPCR Mix; 4 μl cDNA and 5 μl Nuclease-free water were then added, and the final volume was 20 μl. The qPCR procedure was as follows: Cycling conditions after initial denaturation 30 s at 95°C: denaturation 5 s at 95°C, annealing/extension 30 s at 60°C, cycled 45 times. Three biological replicates were performed.

## Results

### Genomic variation and population structure

**Figure 1:**
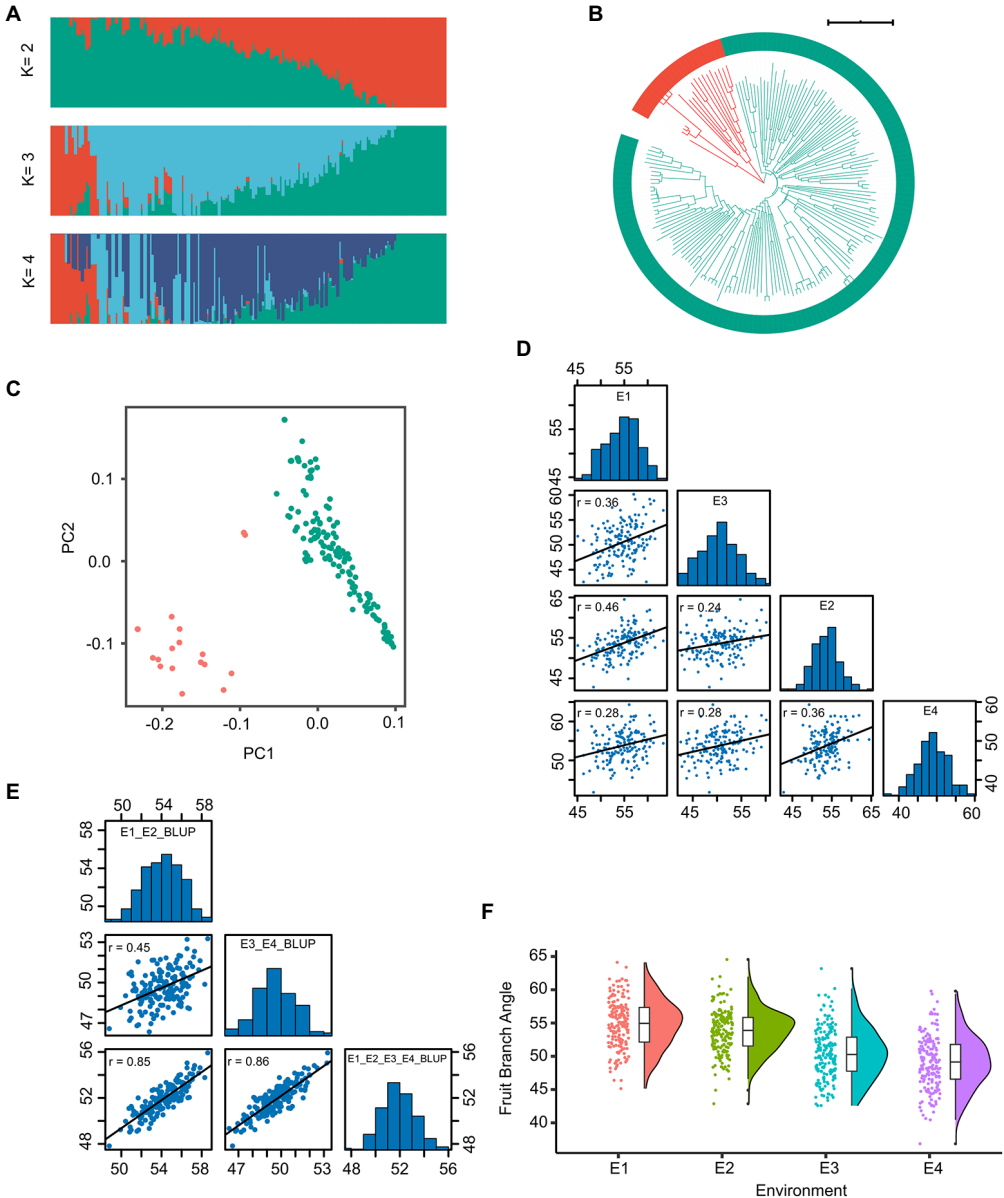
The genotype and phenotype analysis of the 163 cotton accessions. **(A)** Individual ancestry coefficients of 163 cotton accessions determined using ADMIXTURE with the number of ancestry kinships ‘K’ set to 2 or 4. Each accession is denoted by a vertical bar; the proportion of different colors in each bar indicates the proportion of genetic from each of the ancestral populations. **(B)** Phylogenetic tree of the population. **(C)** The plot of principal component analysis (PCA) was shown. **(D)** The histogram and correlation analysis of the frequency distribution of fruit branch angle (FBA) in E1, E2, E3, and E4. **(E)** The histogram and correlation analysis of the frequency distribution of the BLUP of FBA. **(F)** The boxplot of FBA in four environments were shown.

We constructed a high-density map with genetic variation based on re-sequencing data of 163 accessions with an average of 12.12-fold sequencing depth (Supplementary Table S3). In total, 2,499,987 SNPs distributed randomly on 26 chromosomes were identified for the 163-accession panel. The highest density of SNPs was detected on chromosome A01, whereas the lowest density of SNPs was detected on chromosome A02, and the average marker density was 1.16 SNP per kb (Supplementary Table S4). The population structure analysis (Figure 1A), phylogenetic-tree construction (Figure 1B) and PCA (Figure 1C) were performed. The result showed that these accessions were classified into two groups, without obvious difference between the xinluzao-and xinluzhong-named accessions.

### Phenotypic variations in fruit branch angle among accessions

We collected phenotypic data for the association panel in four environments. The FBA varied from 36.86° to 64.56°, the coefficient of variation was constant in different environments and ranged from 6.02% to 8.00% (Table 1). The broad-sense heritability (*H*^2^) of FBA was 68.70% (Table 1). The FBA was influenced by both environment and genotype, with genotype-by-environment interaction influencing FBA (*p* < 0.001; Supplementary Table S5). The Pearson correlation coefficient (*r*) was used to determine correlation of phenotypic data between two environments. The minimum *r* was 0.24 (E2 and E3) and the maximum *r* was 0.46 (E1 and E2; Figure 1D). The trend of FBA was slightly stable between different years in the same location than between different locations in the same years (Figure 1F). The best linear unbiased prediction (BLUP) was estimated across all four environments of FBA, and showed that the correlation coefficients improved significantly (Figure 1E). These results suggest that the FBA in this population might have a relatively broad genetic basis.

**Table 1:** Phenotypic variation of fruit branch angle (FBA) in four environments.

Environments	Min (°)	Max (°)	Mean ± SD (°)	CV (%)	H^2^ (%)
E1	45.14	64.13	54.84 ± 3.63	6.61	68.70
E2	42.88	64.56	53.78 ± 3.24	6.02	
E3	42.59	63.19	50.42 ± 3.84	7.62	
E4	36.86	59.80	49.04 ± 3.92	8.00	

### Genome-wide association study for FBA

**Figure 2:**
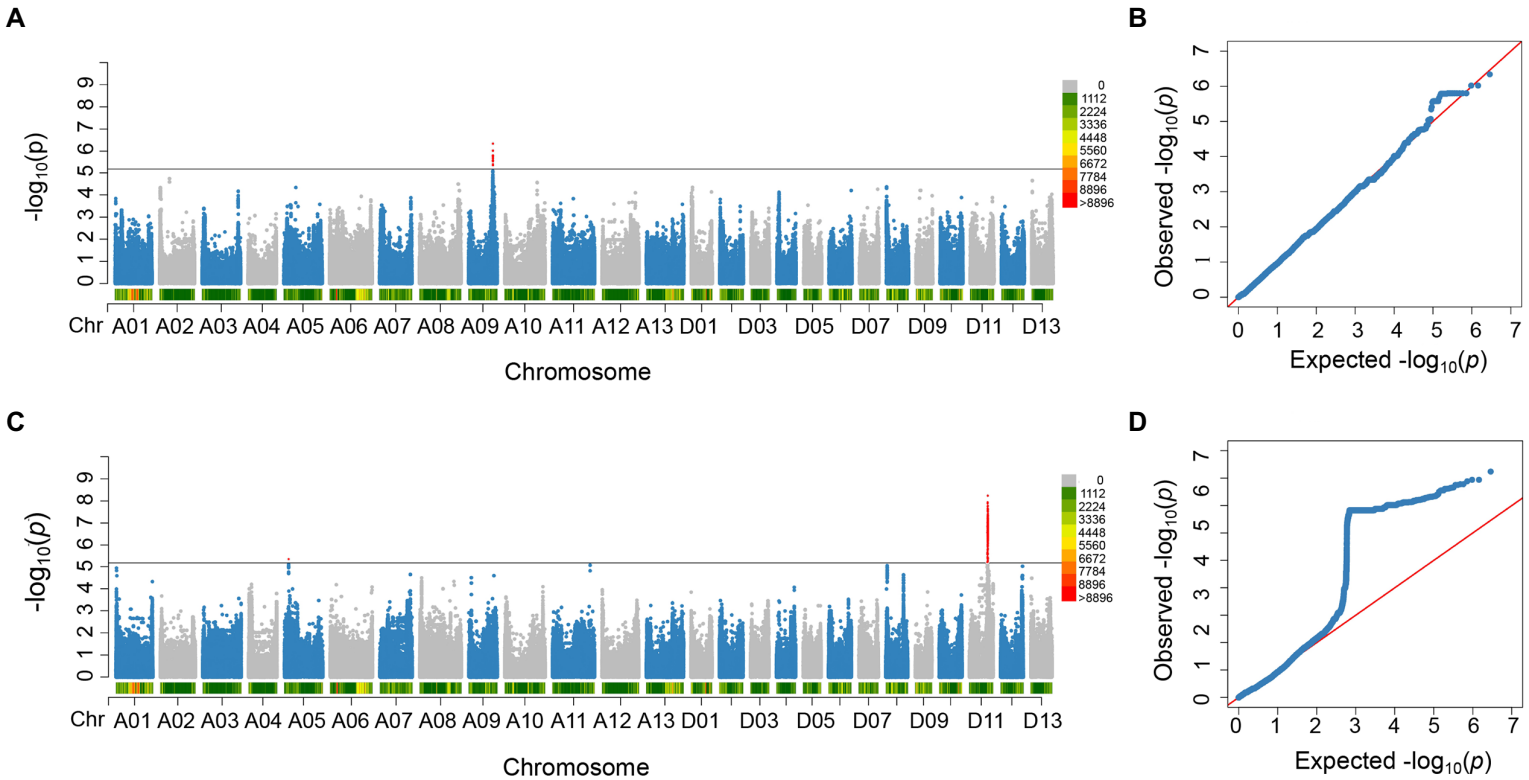
Significant SNPs detected in this study. **(A)** Manhattan plot of the significant SNPs associated with FBA on chromosome A09 in E3_E4_BLUP. **(B)** QQ plot of significant SNPs associated with FBA on chromosome A09. **(C)** Manhattan plots of the significant SNPs associated with FBA on chromosome D11 in E2. **(D)** QQ plot of significant SNPs associated with FBA on chromosome D11.

**Figure 3:**
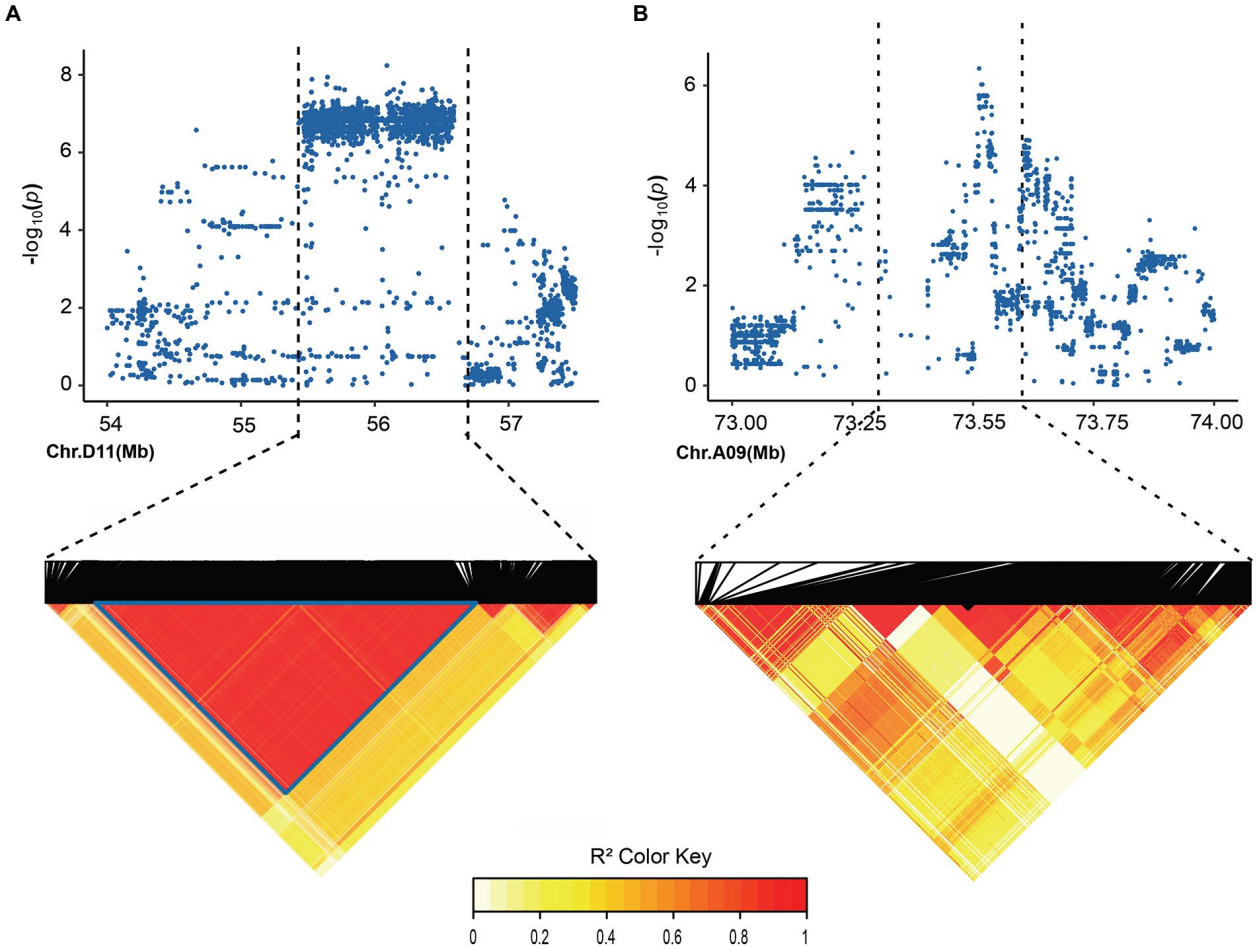
Local Manhattan plot and LD heatmap map. **(A)** local Manhattan plot (top) and LD heatmap map (bottom) surrounding the peak on A09. **(B)** Local Manhattan plot and LD heatmap map surrounding the peak on D11.

In order to identify significant genetic loci and candidate genes related to FBA accurately, different models for GWAS analysis on FBA value in each single environment and the FBA BLUP values in multiple environments were used (Supplementary Figure S1). We applied TASSEL (Q, PCA, K, K + Q), GEMMA (K + Q), EMMAX (K + Q) to perform the association analysis, respectively. We selected GEMMA (K + Q) to perform the association analysis with an indication from the Q–Q plot. The plot was on the theoretical Q–Q line at the start position (Supplementary Figure S1), suggesting that this model was suitable for associating FBA for each single or multiple environments. A sliding window method with default settings for window size of 50 bp-length and step size of 10 bp-length was used to view the average of SNP-index across the whole genome. The number of effective SNPs was 188,048. Herein, the significance threshold was set as: 5.31 × 10^−6^ (*p*-value = 1/188,048). SNPs with *p*-value <5.31 × 10^−6^ were considered as significant SNP-trait associations. GWAS for each environment and BLUP phenotypes were performed. It is found that a total of 55 SNPs with significant association to FBA were identified, with 31 and 24 SNPs on chromosome A09 in two environments and D11 in single environment, respectively (Figure 2; Supplementary Table S6). LD block analysis showed that most peak SNPs were mainly distributed in 73.50–73.54 Mb and 55.40–56.60 Mb on chromosome A09 and D11, respectively (Figures 3A,B). Based on the functional analysis of genes in LD regions, a total of 18 candidate genes were identified for FBA (Table 2).

**Table 2:** Candidate genes in locus FBA and its annotation in GWAS.

Gene ID	Strand	Start	Stop	Annotation
Ghi_D11G13976	−	55606430	55607038	Unkown
Ghi_D11G13986	−	55710171	55710818	Encodes a member of the DREB subfamily A-2 of ERF/AP2 transcription factor family
Ghi_D11G13991	+	55735362	55755871	LITHYIA (ILA) is a HEAT repeat protein involved in plant immunity
Ghi_D11G14006	−	55881903	55884394	Unkown
Ghi_D11G14016	−	55893290	55893505	Distorted trichomes and exhibits a diffuse actin cytoskeleton
Ghi_D11G14021	+	55898226	55899998	Encodes tubulin beta-2/beta-3 chain
Ghi_D11G14026	−	55908280	55910328	Distorted trichomes and exhibits a diffuse actin cytoskeleton
Ghi_D11G14041	−	56367889	56368116	Unkown
Ghi_D11G14046	−	56369536	56369688	Organic solute transporter ostalpha protein (DUF300)
Ghi_D11G14051	+	56442643	56444639	Transmembrane protein
Ghi_D11G14056	+	56469459	56473076	Heme oxygenase-like, multi-helical
Ghi_D11G14061	−	56535658	56536310	Phosphofructokinase family protein
Ghi_A09G08731	+	73510715	73511772	Unkown
Ghi_A09G08736	+	73516570	73516941	SAUR-like auxin-responsive protein family
Ghi_A09G08741	−	73519710	73521047	Transmembrane protein
Ghi_A09G08751	−	73527108	73529504	Encodes a catalytic subunit of the mitochondrially-localized NAD + -dependent isocitrate dehydrogenase
Ghi_A09G08746	−	73523022	73525968	Encodes adenosine kinase 2 (ADK2), a typical, constitutively expressed housekeeping enzyme
Ghi_A09G08756	−	73541451	73543957	Encodes PAL1, a phenylalanine ammonia-lyase

### RNA sequencing of two cotton cultivars with different FBA phenotypes

**Figure 4:**
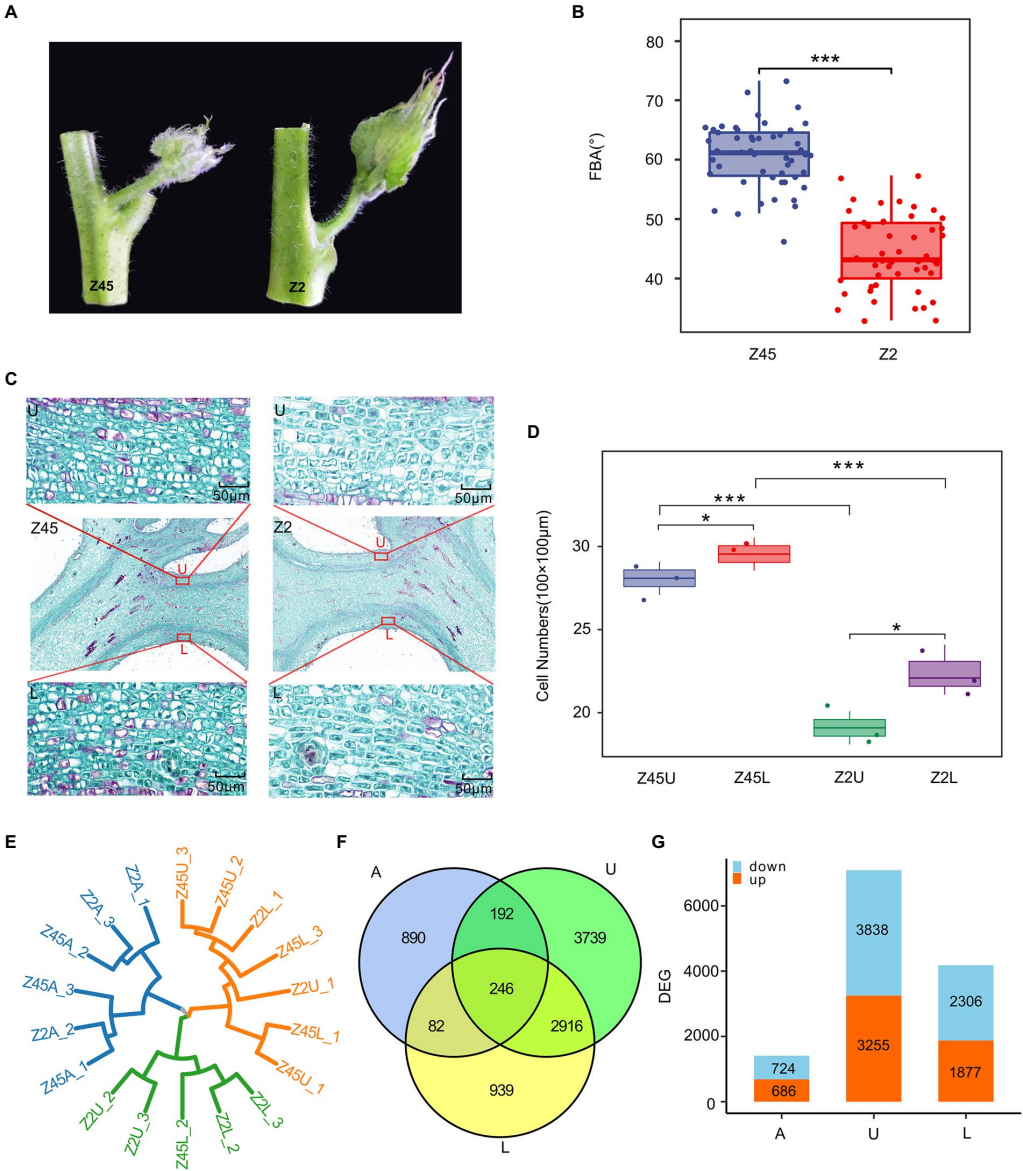
Transcriptome analysis of differentially expressed genes (DEGs) in different samples. **(A)** The FBA differences between Z45 and Z2. **(B)** The FBA of the Z2 and Z45 were shown by using boxplot. **(C)** Histological graph of the parenchyma cells in endodermis of Z2 and Z45 were shown. **(D)** The number of parenchyma cells in endodermis of upper side and lower side of two cultivars were shown. ^*^Significant difference (*p* < 0.05), ^***^Significant difference (*p* < 0.001), *t*-test. **(E)** The cluster dendrogram of all samples were constructed. **(F)** Venn diagrams of DEGs among different sample were shown. **(G)** The numbers of up-regulated and down-regulated gene in different samples were shown by using the histogram.

In order to further explore the genetic basis of FBA formation, two *G. hirsutum* cultivars (Xinluzhong 45 and Xinluzao 2), which are significantly different in the FBA, were selected from the population. The average FBA of Xinluzhong 45 and Xinluzao 2 is 58.80° and 44.40°, respectively (Figures 4A,B). Histological analysis of paraffin sections showed that the number of parenchyma cells in endodermis of upper side, lower side were significantly different in the two cultivars (*p* < 0.001) and between the upper side and lower side of per cultivar (*p* < 0.05) (Figures 4C,D).

To explain the differences in FBA of the fruit branch, the axillary buds (A), upper side (U) and lower side (L) of stem tissue were collected. Then, RNA sequencing was performed on these tissues at different stages of Xinluzhong 45 (Z45) and Xinluzao 2 (Z2) with three biological replicates. A total of 111.77 Gb of clean data were obtained by RNA sequencing. The Q30 reached 94.15%, multiply mapped reads and uniquely mapped reads accounted for 3.58–4.01% and 90.77–94.17% of these reads, respectively (Supplementary Table S7). To assess data reliability, two biological replicates with good reproducibility were selected for differential gene expression analysis (Figure 4E). A total of 1,410, 4,183 and 7,093 differentially expressed genes (DEGs) were identified in A, L and U, and 246 common DEGs were detected in the three tissues (Figure 4F). We found that Z45 has more down-regulated genes than Z2 in three tissues (Figure 4G).

### Gene ontology annotation analysis of DEGs

To uncover the functional implications of 12,686 DEGs, the GO enrichment analysis was performed. The DEGs of all tissues were clustered into 5 categories according to expression patterns. GO enrichment analysis indicated that these five types of DEGs were mainly concentrated in ‘response to gravity’ (GO: 0009629), ‘regulation of actin filament length’ (GO:0030832), ‘response to red or far-red light’ (GO:0009639), ‘flavonoid metabolic process’ (GO:0009812), ‘gibberellic acid mediated’ (GO:0009937) and ‘abscisic acid-activated signaling pathway’ (GO:0009787; Figure 5). Previous studies revealed that gravity and light were correlated with branch angle in plants (Waite and Dardick, 2018; Zhang et al., 2018; Li et al., 2019b; Xie et al., 2019). The high expression levels in Z45U and Z45L were detected in the DEGs response to gravity (Supplementary Figure S2). Two of these genes, encoding shoot gravitropism 6 and LAZY1 protein (Supplementary Table S8), are known to influence the branch angle growth and development (Li et al., 2007; Hashiguchi et al., 2014). In addition, three genes response to red and far-red light pathway showed higher expression in Z2U and Z2L (Supplementary Figure S2), which encode phytochrome A-associated F-box protein (two out of three genes) and PIF1-like transcription factor (one out of three genes; Supplementary Table S8). These findings suggest that PAF and PIF may play a key role in regulation of FBA formation in Z2.

**Figure 5:**
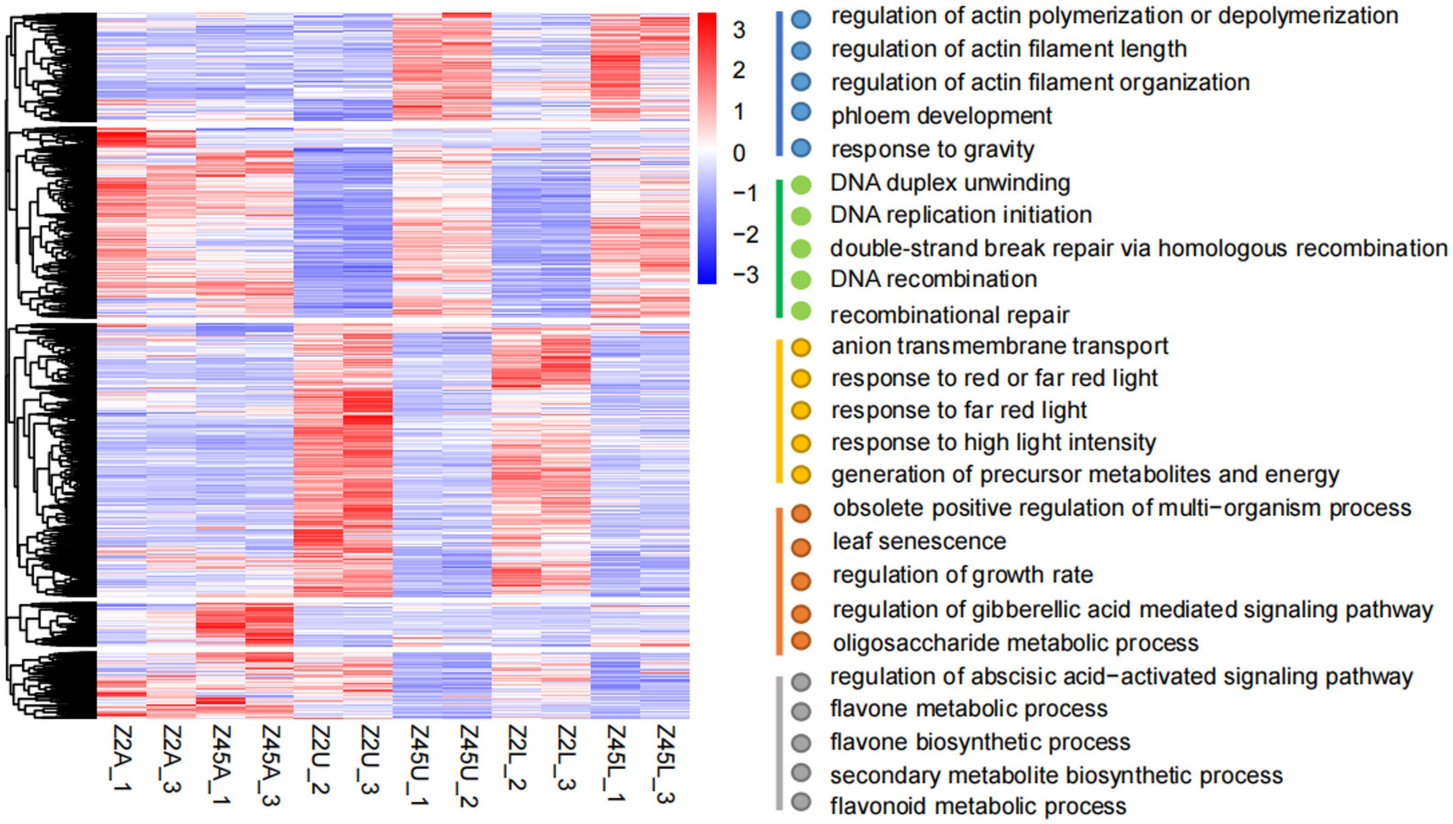
Cluster analysis of 12,686 DEGs. The gene expression pattern of DEGs were shown by heat map, and clustered into five modules. GO enrichment analysis (biological process) was performed on these five modules.

### Discovery of candidate genes for FBA by integrating GWAS and transcriptome data

**Figure 6:**
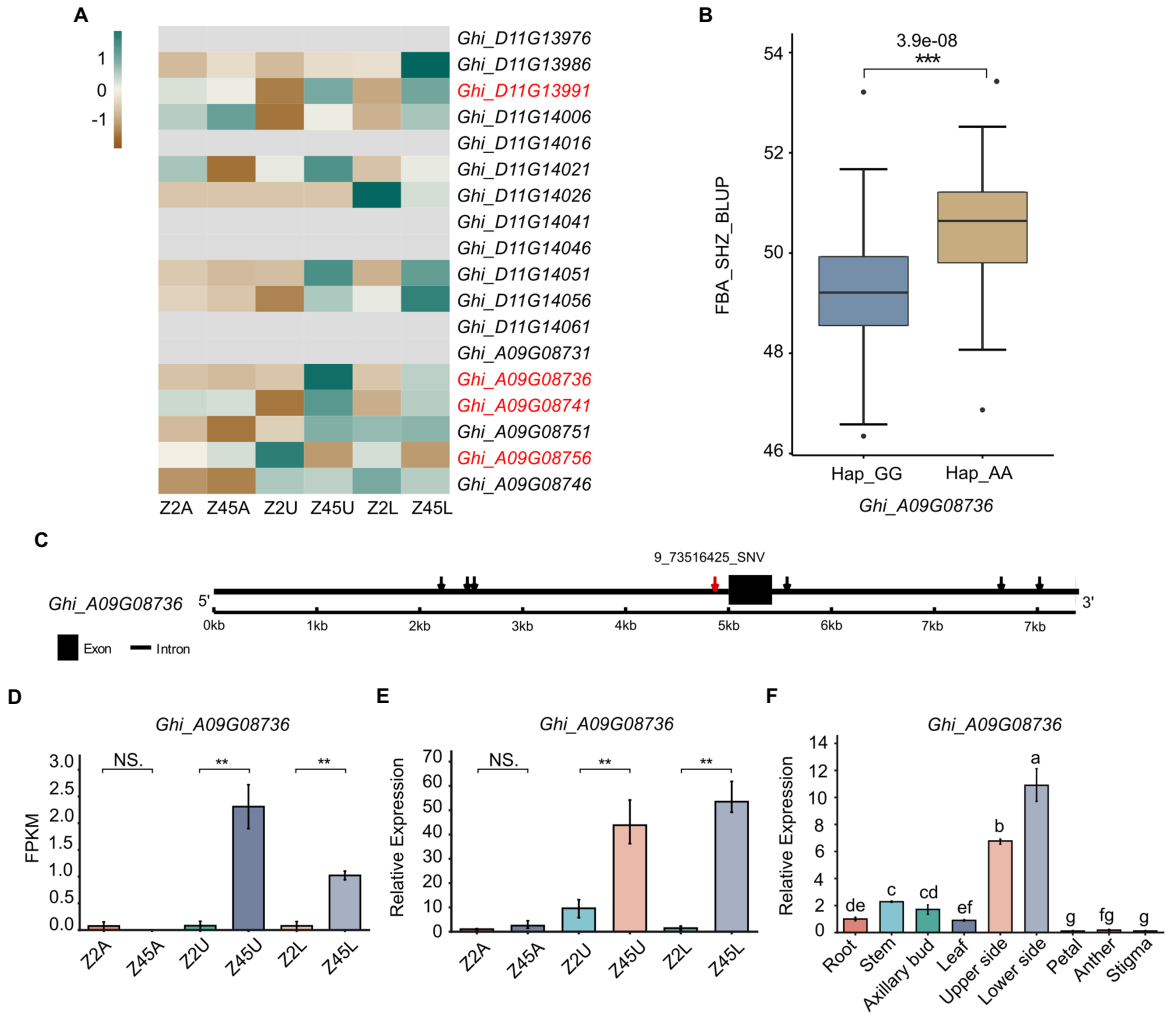
Variation analysis of candidate gene *Ghi_A09G08736* in 163 *Gossypium hirsutum* accessions. **(A)** The expression patterns of 18 candidate genes detected by GWAS were shown by heat map. The red mark of genes indicates the DEGs between Z2 and Z45 detected by RNA-seq. **(B)** Haplotype analysis of the SNP 9_73516425_SNV for *Ghi_A09G08736* were shown. **(C)** Gene structure of *Ghi_A09G08736* was shown. Black rectangles and black lines indicate exon and introns, respectively. **(D)** Gene expression of *Ghi_A09G08736* derived from RNA-seq data. **(E)** Gene expression of *Ghi_A09G08736* derived from qRT-PCR analysis. **(F)** The tissue expression pattern of *Ghi_A09G08736* in TM-1 were shown. **Significant difference (*p* < 0.01), ***Significant difference (*p* < 0.001), *t*-test. a-g mean significant difference (*p* < 0.05), Duncan test.

To further confirm the candidate genes, an analysis that combined GWAS and transcriptome data was performed. In this study, we combined significant association regions, LD block, DEGs and gene annotation to determine the genes related to FBA formation in cotton. Among the 18 genes detected by GWAS, four genes (*Ghi_A09G08736, Ghi_A09G08741, Ghi_A09G08756, Ghi_D11G13991*) were identified from the 12,686 DEGs (Figure 6A). Herein, the four DEGs were predicted as candidates (Figure 6A) and then we performed the expression pattern analysis derived from RNA-seq and qRT-PCR for these candidates (Figures 6D,E; Supplementary Figure S3). It showed that *Ghi_A09G08736* was specifically differentially expressed in the pairwise comparisons (Z2U vs. Z45U and Z2L vs. Z45L) of the large-FBA (Figures 6D,E). To investigate the function of *Ghi_A09G08736* in FBA formation, we performed the qRT-PCR trials at the tissue expression patterns of TM-1. The result showed that *Ghi_A09G08736* was specifically expressed in upper and lower side of fruit branch (Figure 6F). A total of seven SNPs were detected in *Ghi_A09G08736*, and 9_73517125_SNV in the promoter (Figure 6C) and a significant phenotypic difference was revealed between the haplotypes (Hap1_GG, Hap2_AA; Figure 6B). *Ghi_A09G08736* encoding a SAUR-like auxin-responsive protein (Table 2), is key effector output of hormonal and environmental signals in plant growth and development (Ren and Gray, 2015). Therefore, we inferred that *Ghi_A09G08736* may be as an FBA-associated candidate gene in cotton.

## Discussion

### The relationship between FBA and cotton fiber yield

Xinjiang is currently the most dominant cotton-growing region in China, and high planting density are widely adopted to obtain high cotton yield in the region. But high density alone is not enough to establish a high radiation use efficiency population for high cotton yield. The appropriate cotton varieties, such as a relatively compact plant architecture, strong lodging resistance, concentrated flowering and boll-setting, as well as sensitivity to defoliants, were used to improve the cotton yield (Feng et al., 2017). Cotton plant architecture breeding programs have become an effective method for improving yield (Su et al., 2018). Previous studies have shown that branch (leaf or tiller) angle influences yield performance by affecting the efficiency with crop capture light, as well as can influence the productivity and efficiency of mechanical plucking. In sorghum, the angle of upper leaves may be exploited to change light interception and optimize crop canopies for different contexts (Zhi et al., 2022). For tea plants, the branch angle is a critical factor that can greatly influence the productivity and efficiency of mechanical plucking (Xia et al., 2021). As a key factor of cotton plant architecture, the FBA affects plant density, photosynthetic efficiency, disease and lodging resistance, as well as playing an important role in determining cotton fiber yield. Compact cultivars can increase the utilization efficiency of light, water and nutrients, which implies that optimizing FBA can achieve an optimal canopy that is beneficial to light interception and utilization. In this study, a panel consisting of 163 upland cotton accessions was established to study FBA, and it exhibited high phenotypic diversity in the upland cotton population (Table 1). We identified some excellent germplasm resources in FBA for future Chinese-cotton breeding programs.

### QTL mapping of FBA in upland cotton

The genetic control of FBA formation is complex, and is typical of a quantitative trait. The QTL mapping provides an efficient tool for dissecting the genetic basis of FBA, and is useful in improving cotton FBA trait. An interspecific BC_1_ population between *G. hirsutum* and *Gossypium barbadense* was used, and identified 11 epistatic QTLs for FBA trait (Song and Zhang, 2009). Two mapping generations/populations F_2_ and F_2:3_ in upland cotton, derived from ‘Baimian 1’ and TM-1, were used and detected five QTLs (Li et al., 2014).

An association mapping panel comprising 172 *G. hirsutum* accessions was first characterized with a total of 101 polymorphic SSR markers by GWAS for FBA trait. The synergistic alleles and the negative alleles for FBA and some representative accessions can be utilized in plant architecture breeding (Li et al., 2015). Another study identified six associated genetic loci on chromosome A07 for FBA trait *via* GWAS of 355 accessions with 93,250 SNPs (Su et al., 2018). In the present study, 163 germplasm of upland cotton were established as an association population. The FBA under different environmental conditions and BLUP were used as phenotypic data, and 188,048 SNPs were used for GWAS. The 55 SNPs associated with FBA on chromosome A09 and D11 were not reported previously. *Dehydration Response Element Binding factor* (*GhDREB1B*) was an important gene controlling cotton plant architecture, through its effects on hormones signal pathways, and over-expression of *GhDREB1B* confers a significant reduction in FBA (Ji et al., 2021). In this study, the phenotype of FBA was measured in four environments, and 18 genes were identified *via* GWAS. The critical pathways associated with FBA and several genes response to gravity and light were detected by RNA-seq. In marker-assisted breeding, these QTLs can be considered to improve FBA trait. To uncover the genetic mechanisms controlling FBA, further analysis should be proceeded by verifying the function of these genes or other efficient strategy.

### Identification of candidate genes for FBA in upland cotton

Integration of GWAS and DEGs, four genes were detected, in which *Ghi_A09G08736* encodes a SAUR-like auxin-responsive protein (Ren and Gray, 2015). Auxin synthesis and transport, small auxin-up RNAs (SAURs) were the largest family of early auxin-response genes, which played a key role in branch angle formation. In rice, overexpressing the *SAUR39* gene increased leaf and tiller angle compared with wild-type plants (Kant et al., 2009). *SAUR10* was discovered to be repressed by the *Arabidopsis* MADS domain factor FRUITFULL (FUL) in stem and inflorescence branches. *SAUR10* has an effect on branch angle as a function of auxin, brassinolide, light conditions and FUL (Bemer et al., 2017). Two SAUR-like early auxin-response genes, *BnaC03g14890D* (*SAUR30*) and *BnaC03g16420D* (*SAUR55*), were considered as key candidate genes controlling branch angle formation in *B. napus* (Shen et al., 2018). In this study, we identified the gene *Ghi_A09G08736 via* GWAS and RNA-seq, which is homologous to *SAUR46* in *Arabidopsis*. We speculated that *Ghi_A09G08736* also likely influenced auxin synthesis and transport, leading to auxin redistribution and thus regulating the FBA in cotton. The expression pattern derived from RNA-seq and qRT-PCR showed that *Ghi_A09G08736* was specifically differentially expressed in the pairwise comparisons (Z2U vs. Z45U and Z2L vs. Z45L) of the large-FBA (Figures 6D,E) and specifically expressed in upper and lower side of fruit branch by analyzing tissue expression patterns (Figure 6F). These results suggest that *Ghi_A09G08736* may be one of the important genes determining the FBA formation. To confirm the *Ghi_A09G08736* regulating FBA formation *via* auxin synthesis and transport, more studies are needed to further analyze this candidate gene and validate its function in future. Furthermore, in our RNA-seq analysis, we found that the DEGs were significantly response to gravity and light (Figure 5). Further analysis about the genes which were response to gravity allowed us to identify a homology LAZY1 gene (*Ghi_A05G19901*; Supplementary Table S8). Accumulated evidence indicates that *LAZY1* plays a crucial role in plant responses to gravitropism and the branch angle formation (Yoshihara et al., 2013; Yoshihara and Spalding, 2017; Zhu et al., 2020). *AtLAZY1* controls the rice tiller angle by regulating the asymmetric distribution of auxin (Li et al., 2007; Zhang et al., 2018). *CsLAZY1* plays an important role in regulating shoot gravitropism and affecting the branch angle in tea plants, and the over-expression of *CsLAZY1* in *Arabidopsis thaliana* showed that plants responded more effectively to gravity processing under light and dark conditions than did the wild type (Xia et al., 2021). Therefore, in our study, *Ghi_A05G19901* may play roles in response to gravitropism and alter the FBA by acting on the transportation of phytohormones.

## Conclusion

In this study, we combined GWAS and RNA-seq to dissect the genetic basis of FBA trait in cotton. Through GWAS, candidate genes significantly associated with FBA were identified on chromosome A09 and D11, then combined with the DEGs from RNA-seq for further analysis. Four candidate genes were identified. Among them, *Ghi_A09G08736* homologous to *SAUR46* in *Arabidopsis* was detected as an FBA-associated candidate gene in cotton. Meanwhile, key genes and pathways involved in the process of fruit branch development were identified by RNA-seq. This study provides new insights for further dissection of the genetic basis of FBA and helps to dissect the molecular mechanism of the FBA in cotton.

## Data availability statement

The data presented in the study are deposited in the Sequence Read Archive (SRA) repository, accession number PRJNA874737.

## Author contributions

XN and MW designed and supervised the research. YW, XN, LL and MW revised the manuscript. ZP, YY, CY, QY, and XZ guided the content of the article. PS, NA, GS, CG, LC, LL, and XT investigated phenotypic of FBA in Korla and Shihezi. PS and QA collected the sample for RNA-seq. PS, JW, and YP performed the data analysis. PS and YP finished the writing of the manuscript. All authors contributed to the article and approved the submitted version.

## Funding

This work was supported by the Fund for National Natural Science Foundation of China (no. 3206150567), the Innovation Leadership Program in Sciences and Technologies for Young and Middle-aged Scientists of Xinjiang production and Construction Corps, China (no. 2021CB028), Corps science and technology innovation talent plan-Science and Technology Commissioner (no. S2020CB1877), and the Key Programs for Science and Technology Development of Shihezi city, Xinjiang production and Construction Crops, China (no. 2022NY01).

## Conflict of interest

The authors declare that the research was conducted in the absence of any commercial or financial relationships that could be construed as a potential conflict of interest.

## Publisher’s note

All claims expressed in this article are solely those of the authors and do not necessarily represent those of their affiliated organizations, or those of the publisher, the editors and the reviewers. Any product that may be evaluated in this article, or claim that may be made by its manufacturer, is not guaranteed or endorsed by the publisher.

## Footnotes

^1^ https://www.ncbi.nlm.nih.gov/tools/primer-blast/index.cgi?LINK_LOC=BlastHome

## References

[ref1] Bai F. Reinheimer R. Durantini D. Kellogg E. A. Schmidt R. J. (2012). TCP transcription factor, BRANCH ANGLE DEFECTIVE 1 (BAD1), is required for normal tassel branch angle formation in maize. Proc. Natl. Acad. Sci. U. S. A. 109, 12225–12230. doi: 10.1073/pnas.1202439109 22773815 PMC3409762

[ref2] Bemer M. van Mourik H. Muino J. M. Ferrandiz C. Kaufmann K. Angenent G. C. (2017). FRUITFULL controls SAUR10 expression and regulates Arabidopsis growth and architecture. J. Exp. Bot. 68, 3391–3403. doi: 10.1093/jxb/erx184 28586421 PMC5853401

[ref3] Chen Z. J. Scheffler B. E. Dennis E. Triplett B. A. Zhang T. Guo W.. (2007). Toward sequencing cotton (Gossypium) genomes. Plant Physiol. 145, 1303–1310. doi: 10.1104/pp.107.107672 18056866 PMC2151711

[ref4] Chen S. Zhou Y. Chen Y. Gu J. (2018). Fastp: an ultra-fast all-in-one FASTQ preprocessor. Bioinformatics 34, i884–i890. doi: 10.1093/bioinformatics/bty560 30423086 PMC6129281

[ref5] Dieterle M. Zhou Y. C. Schafer E. Funk M. Kretsch T. (2001). EID1, an F-box protein involved in phytochrome A-specific light signaling. Genes Dev. 15, 939–944. doi: 10.1101/gad.197201 11316788 PMC312672

[ref6] Dong H. Zhao H. Li S. Han Z. Hu G. Liu C.. (2018). Genome-wide association studies reveal that members of bHLH subfamily 16 share a conserved function in regulating flag leaf angle in rice (Oryza sativa). PLoS Genet. 14:e1007323. doi: 10.1371/journal.pgen.1007323 29617374 PMC5902044

[ref7] Dong H. Zhao H. Xie W. Han Z. Li G. Yao W.. (2016). A novel tiller angle gene, TAC3, together with TAC1 and D2 largely determine the natural variation of tiller angle in rice cultivars. PLoS Genet. 12:e1006412. doi: 10.1371/journal.pgen.1006412 27814357 PMC5096673

[ref8] Feng L. Dai J. Tian L. Zhang H. Li W. Dong H. (2017). Review of the technology for high-yielding and efficient cotton cultivation in the northwest inland cotton-growing region of China. Field Crop Res. 208, 18–26. doi: 10.1016/j.fcr.2017.03.008

[ref9] Fukaki H. Fujisawa H. Tasaka M. (1996). SGR1, SGR2, and SGR3: novel genetic loci involved in shoot gravitropism in *Arabidopsis thaliana*. Plant Physiol. 110, 945–955. 8819871 10.1104/pp.110.3.945 PMC157794

[ref10] Hashiguchi Y. Yano D. Nagafusa K. Kato T. Saito C. Uemura T.. (2014). A unique HEAT repeat-containing protein SHOOT GRAVITROPISM6 is involved in vacuolar membrane dynamics in gravity-sensing cells of Arabidopsis inflorescence stem. Plant Cell Physiol. 55, 811–822. doi: 10.1093/pcp/pcu020 24486761 PMC3982123

[ref11] Huang G. Hu H. van de Meene A. Zhang J. Dong L. Zheng S.. (2021a). AUXIN RESPONSE FACTORS 6 and 17 control the flag leaf angle in rice by regulating secondary cell wall biosynthesis of lamina joints. Plant Cell 33, 3120–3133. doi: 10.1093/plcell/koab175 34245297 PMC8462825

[ref12] Huang L. Wang W. Zhang N. Cai Y. Liang Y. Meng X.. (2021b). LAZY2 controls rice tiller angle through regulating starch biosynthesis in gravity-sensing cells. New Phytol. 231, 1073–1087. doi: 10.1111/nph.17426 34042184

[ref13] Huang G. Wu Z. Percy R. G. Bai M. Li Y. Frelichowski J. E.. (2020). Genome sequence of Gossypium herbaceum and genome updates of Gossypium arboreum and *Gossypium hirsutum* provide insights into cotton A-genome evolution. Nat. Genet. 52, 516–524. doi: 10.1038/s41588-020-0607-4 32284579 PMC7203013

[ref14] Ji G. Liang C. Cai Y. Pan Z. Meng Z. Li Y.. (2021). A copy number variant at the HPDA-D12 locus confers compact plant architecture in cotton. New Phytol. 229, 2091–2103. doi: 10.1111/nph.17059 33129229

[ref15] Kant S. Bi Y. M. Zhu T. Rothstein S. J. (2009). SAUR39, a small auxin-up RNA gene, acts as a negative regulator of auxin synthesis and transport in rice. Plant Physiol. 151, 691–701. doi: 10.1104/pp.109.143875 19700562 PMC2754634

[ref16] Li C. Q. Ai N. J. Zhu Y. J. Wang Y. Q. Chen X. D. Li F.. (2015). Association mapping and favourable allele exploration for plant architecture traits in upland cotton (*Gossypium hirsutum* L.) accessions. J. Agric. Sci. 154, 567–583. doi: 10.1017/s0021859615000428

[ref17] Li H. Durbin R. (2009). Fast and accurate short read alignment with burrows-wheeler transform. Bioinformatics 25, 1754–1760. doi: 10.1093/bioinformatics/btp324 19451168 PMC2705234

[ref18] Li H. Durbin R. (2010). Fast and accurate long-read alignment with burrows-wheeler transform. Bioinformatics 26, 589–595. doi: 10.1093/bioinformatics/btp698, PMID: 20080505 PMC2828108

[ref19] Li H. Li J. Song J. Zhao B. Guo C. Wang B.. (2019a). An auxin signaling gene BnaA3.IAA7 contributes to improved plant architecture and yield heterosis in rapeseed. New Phytol. 222, 837–851. doi: 10.1111/nph.15632, PMID: 30536633

[ref20] Li Z. Liang Y. Yuan Y. Wang L. Meng X. Xiong G.. (2019b). OsBRXL4 regulates shoot Gravitropism and Rice tiller angle through affecting LAZY1 nuclear localization. Mol. Plant 12, 1143–1156. doi: 10.1016/j.molp.2019.05.014 31200078

[ref21] Li C.-Q. Song L. Zhao H.-H. Wang Q.-L. Fu Y.-Z. Jenkins J. (2014). Identification of quantitative trait loci with main and epistatic effects for plant architecture traits in upland cotton (*Gossypium hirsutum* L.). Plant Breed. 133, 390–400. doi: 10.1111/pbr.12161

[ref22] Li P. Wang Y. Qian Q. Fu Z. Wang M. Zeng D.. (2007). LAZY1 controls rice shoot gravitropism through regulating polar auxin transport. Cell Res. 17, 402–410. doi: 10.1038/cr.2007.38 17468779

[ref23] Li H. Zhang L. Hu J. Zhang F. Chen B. Xu K.. (2017). Genome-wide association mapping reveals the genetic control underlying branch angle in rapeseed (Brassica napus L.). Front. Plant Sci. 8:1054. doi: 10.3389/fpls.2017.01054 28674549 PMC5474488

[ref24] Livak K. J. Schmittgen T. D. (2001). Analysis of relative gene expression data using real-time quantitative PCR and the 2(-Delta Delta C(T)) method. Methods 25, 402–408. doi: 10.1006/meth.2001.1262 11846609

[ref25] Love M. I. Huber W. Anders S. (2014). Moderated estimation of fold change and dispersion for RNA-seq data with DESeq2. Genome Biol. 15:550. doi: 10.1186/s13059-014-0550-8 25516281 PMC4302049

[ref26] Maldonado C. Mora F. Scapim C. A. Coan M. (2019). Genome-wide haplotype-based association analysis of key traits of plant lodging and architecture of maize identifies major determinants for leaf angle: hapLA4. PLoS One 14:e0212925. doi: 10.1371/journal.pone.0212925 30840677 PMC6402688

[ref27] McKenna A. Hanna M. Banks E. Sivachenko A. Cibulskis K. Kernytsky A.. (2010). The genome analysis toolkit: a MapReduce framework for analyzing next-generation DNA sequencing data. Genome Res. 20, 1297–1303. doi: 10.1101/gr.107524.110 20644199 PMC2928508

[ref28] Morita M. T. Sakaguchi K. Kiyose S. Taira K. Kato T. Nakamura M.. (2006). A C2H2-type zinc finger protein, SGR5, is involved in early events of gravitropism in Arabidopsis inflorescence stems. Plant J. 47, 619–628. doi: 10.1111/j.1365-313X.2006.02807.x 16813575

[ref29] Nakamura M. Toyota M. Tasaka M. Morita M. T. (2011). An Arabidopsis E3 ligase, SHOOT GRAVITROPISM9, modulates the interaction between statoliths and F-actin in gravity sensing. Plant Cell 23, 1830–1848. doi: 10.1105/tpc.110.079442 21602290 PMC3123953

[ref30] Nie X. Wen T. Shao P. Tang B. Nuriman-Guli A. Yu Y.. (2020). High-density genetic variation maps reveal the correlation between asymmetric interspecific introgressions and improvement of agronomic traits in upland and Pima cotton varieties developed in Xinjiang, China. Plant J. 103, 677–689. doi: 10.1111/tpj.14760 32246786 PMC7496985

[ref31] Pendleton J. W. Smith G. E. Winter S. R. Johnston T. J. (1968). Field investigation of the relationships of leaf angle in corn (*Zea mays* L.) to grain yield and apparent photosynthesis. Agron. J. 60, 422–424.

[ref32] Pritchard J. K. Stephens M. Donnelly P. (2000). Inference of population structure using multilocus genotype data. Genetics 155, 945–959. doi: 10.1093/genetics/155.2.945 10835412 PMC1461096

[ref33] Rakusova H. Abbas M. Han H. Song S. Robert H. S. Friml J. (2016). Termination of shoot Gravitropic responses by auxin feedback on PIN3 polarity. Curr. Biol. 26, 3026–3032. doi: 10.1016/j.cub.2016.08.067 27773568

[ref34] Ren H. Gray W. M. (2015). SAUR proteins as effectors of hormonal and environmental signals in plant growth. Mol. Plant 8, 1153–1164. doi: 10.1016/j.molp.2015.05.003 25983207 PMC5124491

[ref35] Shen Y. Yang Y. Xu E. Ge X. Xiang Y. Li Z. (2018). Novel and major QTL for branch angle detected by using DH population from an exotic introgression in rapeseed (*Brassica napus* L.). Theor. Appl. Genet. 131, 67–78. doi: 10.1007/s00122-017-2986-1 28942459

[ref36] Shina J. Kim K. Kang H. Zulfugarov I. S. Bae G. Lee C.-H.. (2009). Phytochromes promote seedling light responses by inhibiting four negatively-acting phytochrome-interacting factors. PANS 106, 7660–7665. doi: 10.1073/pnas.0812219106 PMC2678665 19380720

[ref37] Song X. Zhang T. (2009). Quantitative trait loci controlling plant architectural traits in cotton. Plant Sci. 177, 317–323. doi: 10.1016/j.plantsci.2009.05.015

[ref38] Su J. Li L. Zhang C. Wang C. Gu L. Wang H.. (2018). Genome-wide association study identified genetic variations and candidate genes for plant architecture component traits in Chinese upland cotton. Theor. Appl. Genet. 131, 1299–1314. doi: 10.1007/s00122-018-3079-5 29497767

[ref39] Sun C. Wang B. Wang X. Hu K. Li K. Li Z.. (2016). Genome-wide association study dissecting the genetic architecture underlying the branch angle trait in rapeseed (*Brassica napus* L.). Sci. Rep. 6:33673. doi: 10.1038/srep33673 27646167 PMC5028734

[ref40] Tian F. Bradbury P. J. Brown P. J. Hung H. Sun Q. Flint-Garcia S.. (2011). Genome-wide association study of leaf architecture in the maize nested association mapping population. Nat. Genet. 43, 159–162. doi: 10.1038/ng.746 21217756

[ref41] Waite J. M. Dardick C. (2018). TILLER ANGLE CONTROL 1 modulates plant architecture in response to photosynthetic signals. J. Exp. Bot. 69, 4935–4944. doi: 10.1093/jxb/ery253 30099502

[ref42] Wang Y. Li J. (2008a). Molecular basis of plant architecture. Annu. Rev. Plant Biol. 59, 253–279. doi: 10.1146/annurev.arplant.59.032607.092902 18444901

[ref43] Wang Y. Li J. (2008b). Rice, rising. Nat. Genet. 40, 1273–1275. doi: 10.1016/j.mrfmmm.2008.08.008 18957983

[ref44] Wu X. Tang D. Li M. Wang K. Cheng Z. (2013). *Loose plant Architecture1*, an INDETERMINATE DOMAIN protein involved in shoot gravitropism, regulates plant architecture in rice. Plant Physiol. 161, 317–329. doi: 10.1104/pp.112.208496 23124325 PMC3532263

[ref45] Xia X. Mi X. Jin L. Guo R. Zhu J. Xie H.. (2021). CsLAZY1 mediates shoot gravitropism and branch angle in tea plants (Camellia sinensis). BMC Plant Biol. 21:243. doi: 10.1186/s12870-021-03044-z 34049485 PMC8164267

[ref46] Xie C. Zhang G. An L. Chen X. Fang R. (2019). Phytochrome-interacting factor-like protein OsPIL15 integrates light and gravitropism to regulate tiller angle in rice. Planta 250, 105–114. doi: 10.1007/s00425-019-03149-8 30927053

[ref47] Yamauchi Y. Fukaki H. Fujisawa H. Tasaka M. (1997). Mutations in the SGR4, SGR5 and SGR6 loci of *Arabidopsis thaliana* alter the shoot gravitropism. Plant Cell Physiol. 38, 530–535. doi: 10.1093/oxfordjournals.pcp.a029201 9210330

[ref48] Yoshihara T. Spalding E. P. (2017). LAZY genes mediate the effects of gravity on auxin gradients and plant architecture. Plant Physiol. 175, 959–969. doi: 10.1104/pp.17.00942 28821594 PMC5619908

[ref49] Yoshihara T. Spalding E. P. Iino M. (2013). AtLAZY1 is a signaling component required for gravitropism of the *Arabidopsis thaliana* inflorescence. Plant J. 74, 267–279. doi: 10.1111/tpj.12118 23331961

[ref50] Zhang S. Wang S. Xu Y. Yu C. Shen C. Qian Q.. (2015). The auxin response factor, OsARF19, controls rice leaf angles through positively regulating OsGH3-5 and OsBRI1. Plant Cell Environ. 38, 638–654. doi: 10.1111/pce.12397 24995795

[ref51] Zhang N. Yu H. Yu H. Cai Y. Huang L. Xu C.. (2018). A core regulatory pathway controlling rice tiller angle mediated by the LAZY1-dependent asymmetric distribution of auxin. Plant Cell 30, 1461–1475. doi: 10.1105/tpc.18.00063 29915152 PMC6096585

[ref52] Zhao H. Huai Z. Xiao Y. Wang X. Yu J. Ding G.. (2014). Natural variation and genetic analysis of the tiller angle gene MsTAC1 in *Miscanthus sinensis*. Planta 240, 161–175. doi: 10.1007/s00425-014-2070-x 24771021

[ref53] Zhi X. Tao Y. Jordan D. Borrell A. Hunt C. Cruickshank A.. (2022). Genetic control of leaf angle in sorghum and its effect on light interception. J. Exp. Bot. 73, 801–816. doi: 10.1093/jxb/erab467 34698817

[ref340] Zhou X. Stephens M. (2012). Genome-wide efficient mixed-model analysis for association studies. Nat Genet. 44, 821–824. doi: 10.1038/ng.2310 22706312 PMC3386377

[ref54] Zhu M. Hu Y. Aizi T. Yan B. Lv Y. Wang S.. (2020). LAZY1 controls tiller angle and shoot Gravitropism by regulating the expression of auxin transporters and signaling factors in rice. Plant Cell Physiol. 61, 2111–2125. doi: 10.1093/pcp/pcaa131 33067639

