# Supplementary material for: Genome-wide association study and transcriptome analysis reveal key genes controlling fruit branch angle in cotton
Source: Front Plant Sci. 2022 Sep 21;13:988647. doi: 10.3389/fpls.2022.988647 (PMC9532966; doi:10.3389/fpls.2022.988647)
Supplement: Supplementary file 1 [file Data_Sheet_1.docx]

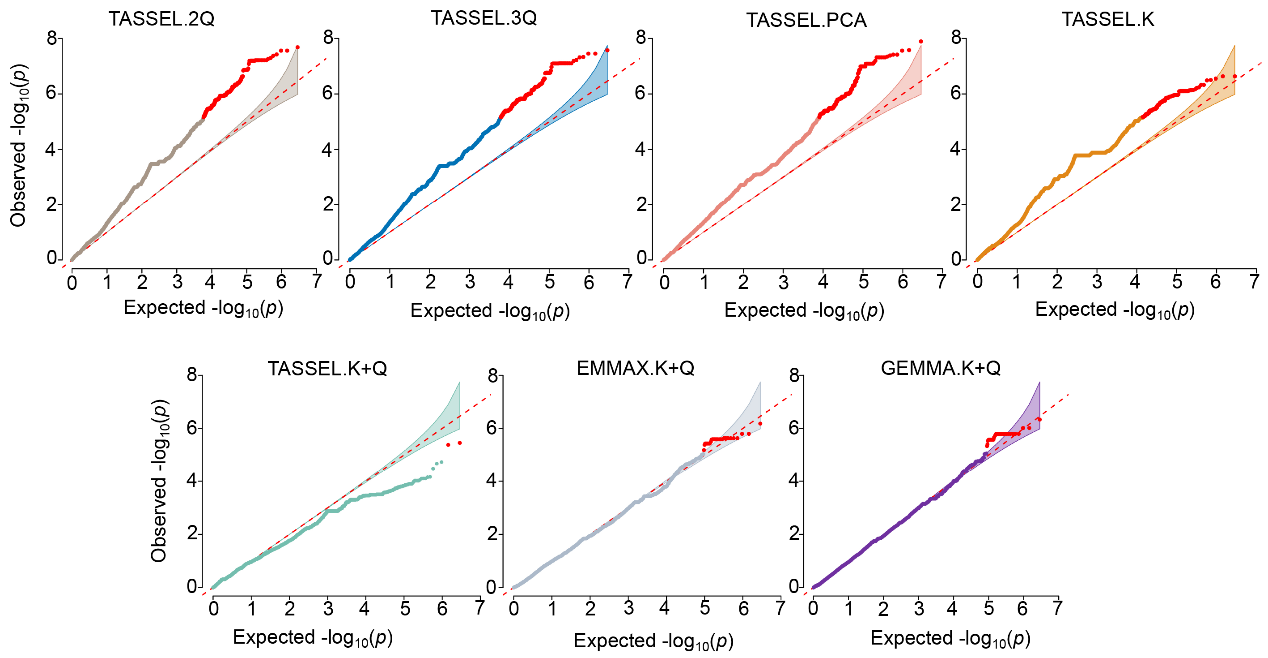


**Figure S1 Q-Q plot of six models for the BLUP of FBA**

**
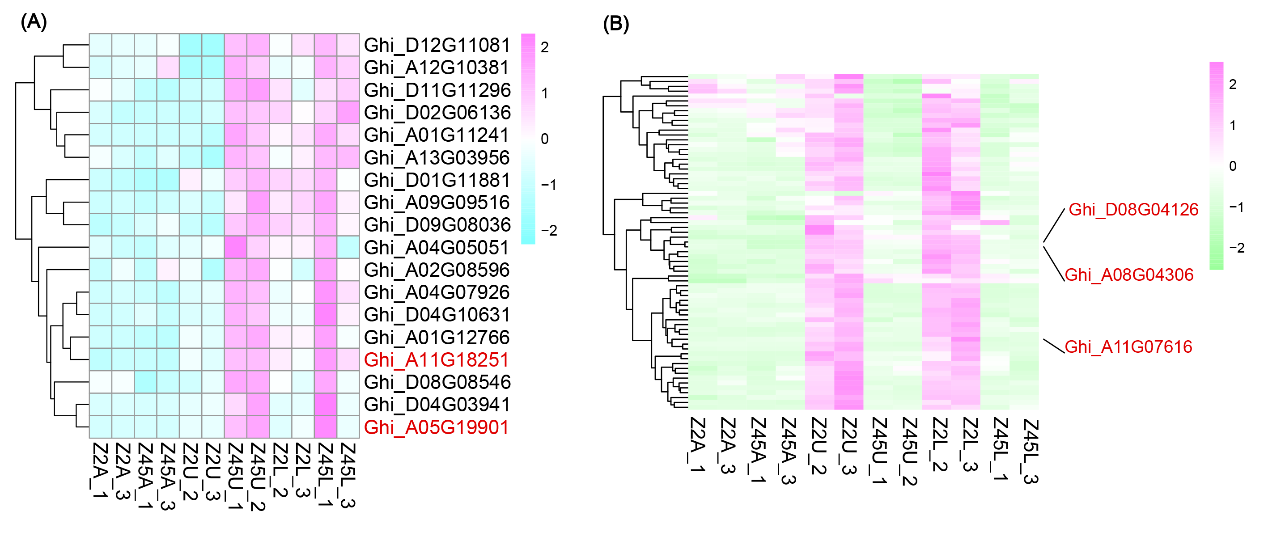
 Figure S2 Analysis of DEGs related to FBA.**

**(A)** Analysis of DEGs which were response to gravity. **(B)** Analysis of DEGs which were response to red and far-red light.

**
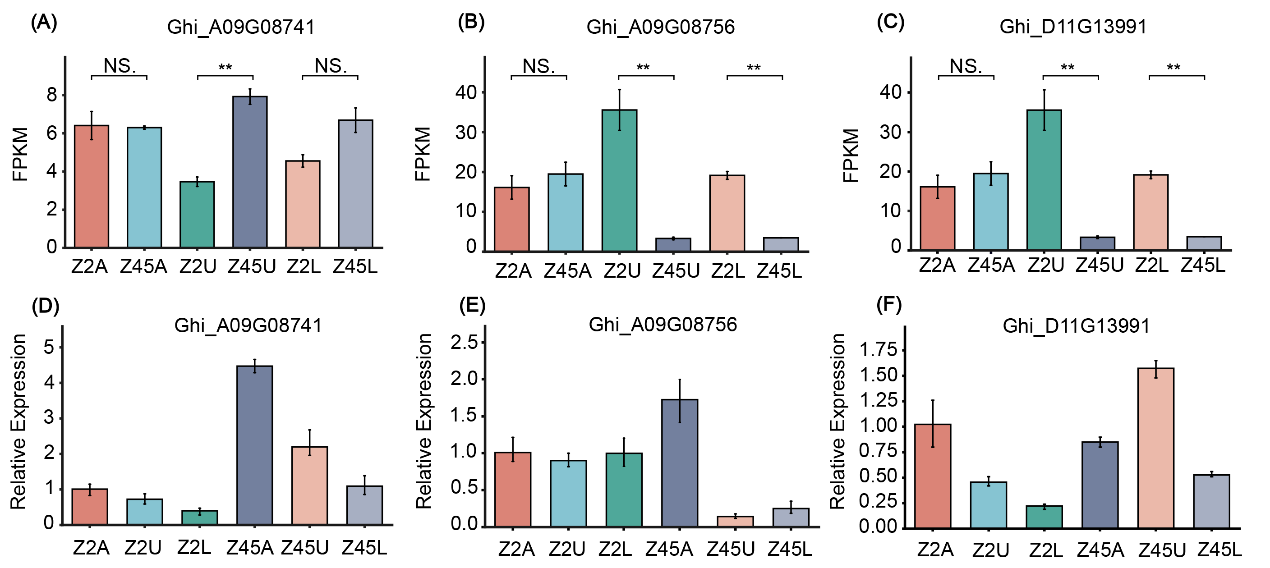
**

**Figure S3 Variation analysis of candidate gene *Ghi_A09G0874, Ghi_A09G08756* and *Ghi_D11G13991***

**(A)** Gene expression of *Ghi_A09G08741* derived from RNA-seq data. **(B)** Gene expression of *Ghi_A09G08756* derived from RNA-seq data. **(C)** Gene expression of *Ghi_D11G13991* derived from RNA-seq data. **(D)** Gene expression of *Ghi_A09G08741* derived from qRT-PCR analysis. **(E)** Gene expression of *Ghi_A09G08756* derived from qRT-PCR analysis. **(F)** Gene expression of *Ghi_D11G13991* derived from qRT-PCR analysis.
